# Axonal Regeneration after Sciatic Nerve Lesion Is Delayed but Complete in GFAP- and Vimentin-Deficient Mice

**DOI:** 10.1371/journal.pone.0079395

**Published:** 2013-11-01

**Authors:** Alexander Berg, Johan Zelano, Marcela Pekna, Ulrika Wilhelmsson, Milos Pekny, Staffan Cullheim

**Affiliations:** 1 Department of Neuroscience, Karolinska Institute, Stockholm, Sweden; 2 Department of Neuroscience, Uppsala University, Uppsala, Sweden; 3 Center for Brain Repair and Rehabilitation, Department of Clinical Neuroscience and Rehabilitation, Institute of Neuroscience and Physiology, Sahlgrenska Academy at the University of Gothenburg, Gothenburg, Sweden; 4 Florey Institute of Neuroscience and Mental Health, Melbourne, Australia; University of Sydney, Australia

## Abstract

Peripheral axotomy of motoneurons triggers Wallerian degeneration of injured axons distal to the lesion, followed by axon regeneration. Centrally, axotomy induces loss of synapses (synaptic stripping) from the surface of lesioned motoneurons in the spinal cord. At the lesion site, reactive Schwann cells provide trophic support and guidance for outgrowing axons. The mechanisms of synaptic stripping remain elusive, but reactive astrocytes and microglia appear to be important in this process. We studied axonal regeneration and synaptic stripping of motoneurons after a sciatic nerve lesion in mice lacking the intermediate filament (nanofilament) proteins glial fibrillary acidic protein (GFAP) and vimentin, which are upregulated in reactive astrocytes and Schwann cells. Seven days after sciatic nerve transection, ultrastructural analysis of synaptic density on the somata of injured motoneurons revealed more remaining boutons covering injured somata in *GFAP^–/–^Vim^–/–^* mice. After sciatic nerve crush in *GFAP^–/–^Vim^–/–^* mice, the fraction of reinnervated motor endplates on muscle fibers of the gastrocnemius muscle was reduced 13 days after the injury, and axonal regeneration and functional recovery were delayed but complete. Thus, the absence of GFAP and vimentin in glial cells does not seem to affect the outcome after peripheral motoneuron injury but may have an important effect on the response dynamics.

## Introduction

Schwann cells in the peripheral nervous system and astrocytes in the central nervous system (CNS) are intimately involved in the response to peripheral nerve lesions. Within a few days after peripheral axotomy, Schwann cells actively participate in Wallerian degeneration of severed peripheral axons distal to the lesion site and guide regenerating motor axons to their targets [1], [2]. They also provide trophic support and remyelinate the lesioned axons [3], [4]. Simultaneously, astrocytes are prominently activated in the spinal cord near lesioned motoneurons in the ventral horn. Ultrastructurally, astrocyte processes extend on the surface of the lesioned motoneurons. This coincides with a loss of synapses (synaptic stripping) from the motoneurons [5]-[9], implying astrocyte involvement.

A hallmark of activation of Schwann cells and astrocytes after nerve lesion is the increased expression of glial fibrillary acidic protein (GFAP) and vimentin. Genetic ablation of intermediate filament proteins in mice provides a model for understanding the role of the intermediate filament system in astrocyte activation in various diseases [10]-[15]. After partial hippocampal de-afferentation induced by entorhinal cortex lesion, *GFAP^–/–^Vim^–/–^* mice have a greater loss of synapses in the hippocampus than wildtype (WT) mice at 4 days but a better synaptic recovery later on [11]. During peripheral nerve regeneration, *GFAP^–/–^* mice with normal levels of vimentin have delayed axonal regrowth and impaired proliferation of Schwann cells [16]. Vimentin is expressed both in non-myelin-forming and myelin-forming Schwann cells [17], [18], and in the absence of both GFAP and vimentin, no intermediate filaments are formed even in reactive astroglial cells [19].Thus, a more severe phenotype could be expected in *GFAP^–/–^Vim^–/–^* mice than in *GFAP^–/–^* mice. The effect of GFAP and/or VIM gene deletion on synaptic stripping after peripheral nerve lesion is unknown.

In this study, we used *GFAP^–/–^Vim^–/–^* mice to investigate whether the intermediate filament proteins GFAP and vimentin participate in synaptic stripping or axon regeneration of motoneurons after axotomy.

## Materials and Methods

### Animal experiments

Adult *GFAP^–/–^Vim^–/–^* (>5 months old ) [20], [21] and age-matched WT female mice were anesthetized with a 2∶1∶1 mixture of water, midazolam (Dormicum, Roche Diagnostics; 1.25 mg/ml), and Hypnorm (Janssen) applied i.p. at 0.2 ml per 20 g of body weight. All mice were on a C57BL/6-129Sv-129Ola genetic background.

The left sciatic nerve was transected or crushed at the obturator tendon level. In the transection model, a 2–4-mm segment of the distal portion of the nerve was removed to prevent regeneration. In the crush model, a pair of forceps was placed around the nerve and a constant pressure was applied for 10 seconds. After 7–33 days, the mice were subjected to lethal carbon dioxide inhalation. All experiments were conducted and approved according to the local ethics committee guidelines ‘Stockholms Norra Försöksdjursetiska nämnd’.

### Immunohistochemistry

Mice used for immunohistochemistry were transcardially perfused with Tyrode’s solution for 30 seconds and then with Lana’s fixative (4% formalin and 0.4% picric acid in 0.1 M PBS, pH 7.2) at 20°C. The spinal cords were rapidly dissected and kept in the same fixative for 90–180 min or overnight at 4°C, rinsed, and stored for 24 h in 10% sucrose with 0.1% sodium azide in 0.01 M PBS at 4°C for cryoprotection. The tissues were cut into sections 14 or 30 µm thick.

Sections were incubated overnight at 4°C with primary antisera (Table 1) in 0.01 M PBS with 5% donkey serum and 0.3% Triton X-100. After rinsing in 0.01 M PBS, the sections were incubated for 60 min with secondary antibodies conjugated to Cy-2, Alexa-488, Cy-3, or Alexa-568 diluted in PBS and 0.3% Triton X-100, rinsed in PBS, and mounted in PBS-glycerol (1:3). For double-labeling experiments, sections were processed as above with additional antibodies. In control experiments, primary or secondary antibodies or both were omitted.

**Table 1:** Primary and secondary antibodies.

Antibodies	Manufacturer	Raised in	Dilution
Primary			
Synaptophysin	Invitrogen	Rabbit	1∶200
Iba1	Wako	Rabbit	1∶1000
GFAP	Dako	Rabbit	1∶200
Vimentin	Abcam	Chicken	1∶200
S100	Dako	Rabbit	1∶400
Neurofilament-200	Sigma-Aldrich	Rabbit	1∶1000
MBP	Millipore	Sheep	1∶1000
α-bungarotoxin	Invitrogen	–	1∶200
Secondary			
Alexa-488, Alexa-568Cy-2 or Cy-3	Invitrogen or The Jackson Laboratories	Donkey	1∶200 (Alexa-488)1∶500 (Alexa-568 and Cy-3)

After immunohistochemistry, sections were examined with a Zeiss LSM 5 Pascal confocal laser scanning microscope (Carl Zeiss GmbH, Göttingen, Germany), equipped with argon/HeNe lasers. Cy-2 was visualized at 488 nm and Cy-3 at 543 nm.

Immunoreactivity (IR) on confocal images of the sciatic motoneuron pool or nerve was measured semiquantitatively with ImageJ (NIH). IR in an area containing the injured sciatic motoneuron pool was compared to an area of equal size containing the contralateral uninjured sciatic motoneuron pool in the same spinal cord section. Images were taken in the optical plane with maximal IR, and all settings for compared sections were identical. At least four spinal cord, sciatic nerve, or muscle sections from each mouse were measured. For spinal cords, the mean ipsilateral/contralateral ratio was used for statistical analysis.

For measurement of IR in the nerve, the ratio between pre- and post-lesion sites immunoreactivity was studied. The mean from each mouse was used for statistical analysis.

For assessment of reinnervation of neuromuscular junctions, 14-µm-thick sections of the gastrocnemius muscle were stained with antisera against synaptophysin (1∶200) and α-bungarotoxin (1:200). α-Bungarotoxin-positive motor end-plates were counted and compared to the area counted and to the percentage of synaptophysin-positive motor end-plates.

### Electron microscopy

The mice (3 per group) were transcardially perfused with fixative containing 2% glutaraldehyde in Millonig’s buffer, pH 7.4. The spinal cords were rapidly dissected and kept overnight in the same fixative at 4°C. The specimens were trimmed, osmicated, dehydrated, and embedded. Neurons with large cell bodies (>100 µm in circumference), found in the sciatic motoneuron pool and cut in the nuclear plane were identified as motoneurons by the presence of C-type nerve terminals. Neurons were identified as axotomized based on the occurrence of chromatolytic changes in the cell bodies. Synaptic terminals opposing the motoneuron somata were identified and their number per 100-µm cell membrane length was calculated. Four motoneurons were analyzed in each of three mice of each strain (*GFAP^–/–^Vim^–/–^* and WT mice) (total, 12 motoneurons per strain)

### Behavioral experiments

Behavioral experiments were performed in five mice in each group before and after SNT or SNC. For behavioral experiments, the lesioned paw was painted with water-based paint, and the mouse was placed in a tunnel placed on top of a sheet of paper. When the mouse had traversed the tunnel, the paper was removed, and intermediary toe spread (distance from second to fourth toe) and toe spread (distance from first to fifth toe) were measured from three representative foot prints and averaged. For foot-fault measurements, the mice were placed on top of a cage lid for 60 seconds and footfault frequency was recorded.

For assessment of grip strength, the mice were held by the tail and allowed to grip the cage lid, with the hind limbs in the air. A grip reflex was elicited by placing a metal rod on the soles of the hind feet. The rod was then pulled away until the grip was lost. The injured foot was compared to the intact one. The ability to grip was categorized into three groups: no grip, partial grip, and normal grip. All testing was made by one main observer blinded to genotype and previous results and was confirmed by a similarly blinded co-observer.

### Statistical analysis

GraphPad Prism (version 5.0; GraphPad Software, San Diego, CA, USA) was used for statistical analysis. Gaussian distribution could be assumed for all data, as determined with the Column Statistics function, and the variances were equal, as shown with an F-test. P<0.05 was considered significant. For quantification of immunohistochemistry and in situ hybridization, four spinal cord sections from each mouse were counted, and the mean ipsilateral/contralateral ratio for each mouse was used for statistical analysis. Student’s *t* test was used to compare two groups. One-way ANOVA with Bonferroni’s multiple comparison test was used to compare multiple groups. All analyses were done in a blind fashion. We decided to test our null hypothesis at p<0.05.

## Results

### Upregulation of GFAP and vimentin after nerve lesion

**Figure 1:**
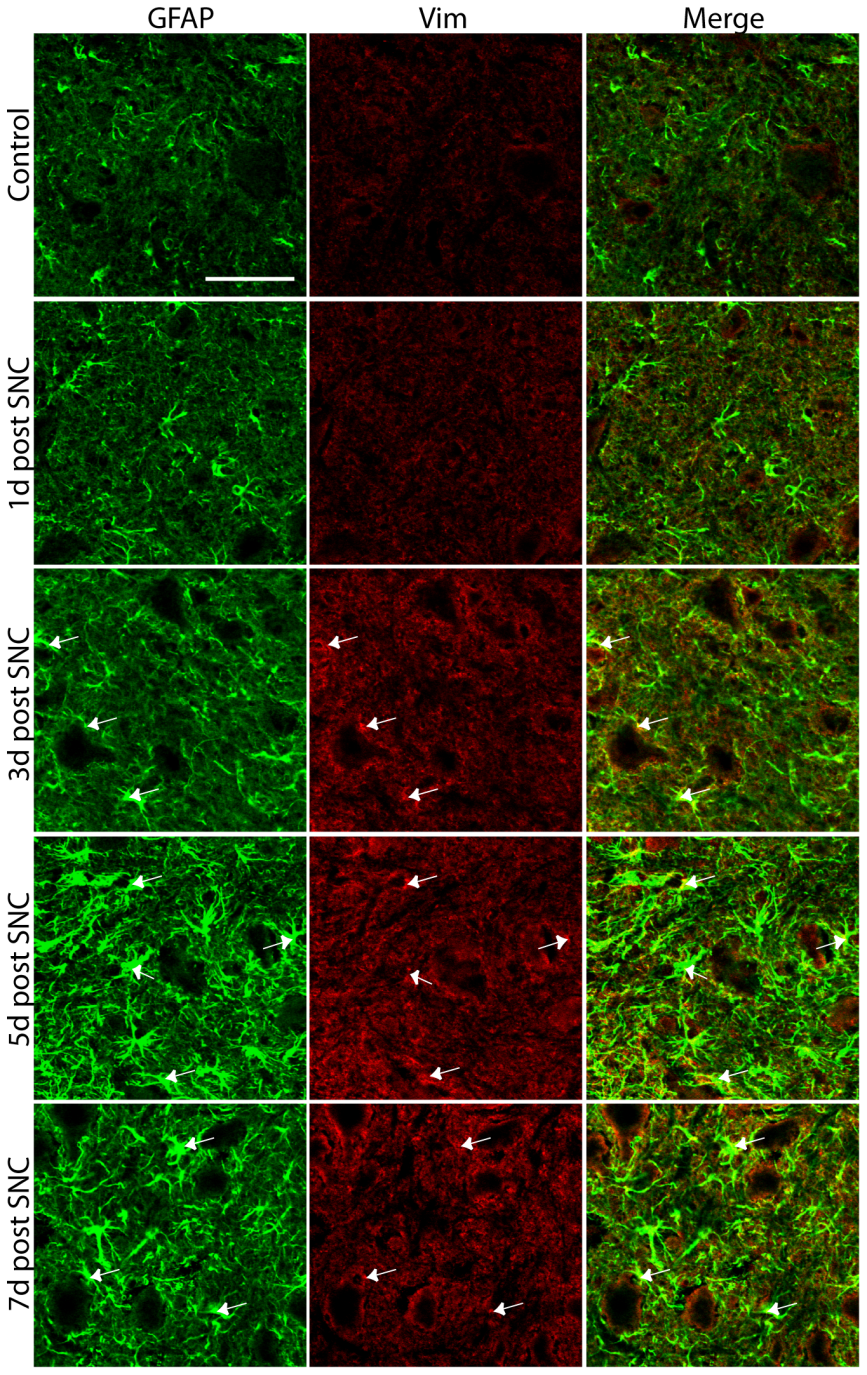
Expression of GFAP and vimentin in the spinal cord. Low levels of GFAP and vimentin (Vim) were detected in the spinal cord before injury. Three days after SNC, the IR for both GFAP and vimentin in the spinal cord surrounding the lesioned motoneurons increased and peaked 5 days after SNC. Arrows indicate co-localization of GFAP and vimentin IR. The picture was taken as a maximal projection and settings were adjusted to optimal levels in a similar fashion for all pictures taken. Scale bar, 50 µm.

After SNT and SNC in WT mice, GFAP and vimentin IR in the spinal cord was strongly upregulated, presumably by reactive and proliferating astrocytes as reported after neurotrauma [22], [23]. The upregulation was most pronounced near injured motoneurons, and the intensity of GFAP and vimentin IR peaked 5 days after SNC (Fig. 1). No GFAP or vimentin IR was seen in the *GFAP^–/–^/Vim^–/–^* mice after injury (data not shown).

**Figure 2:**
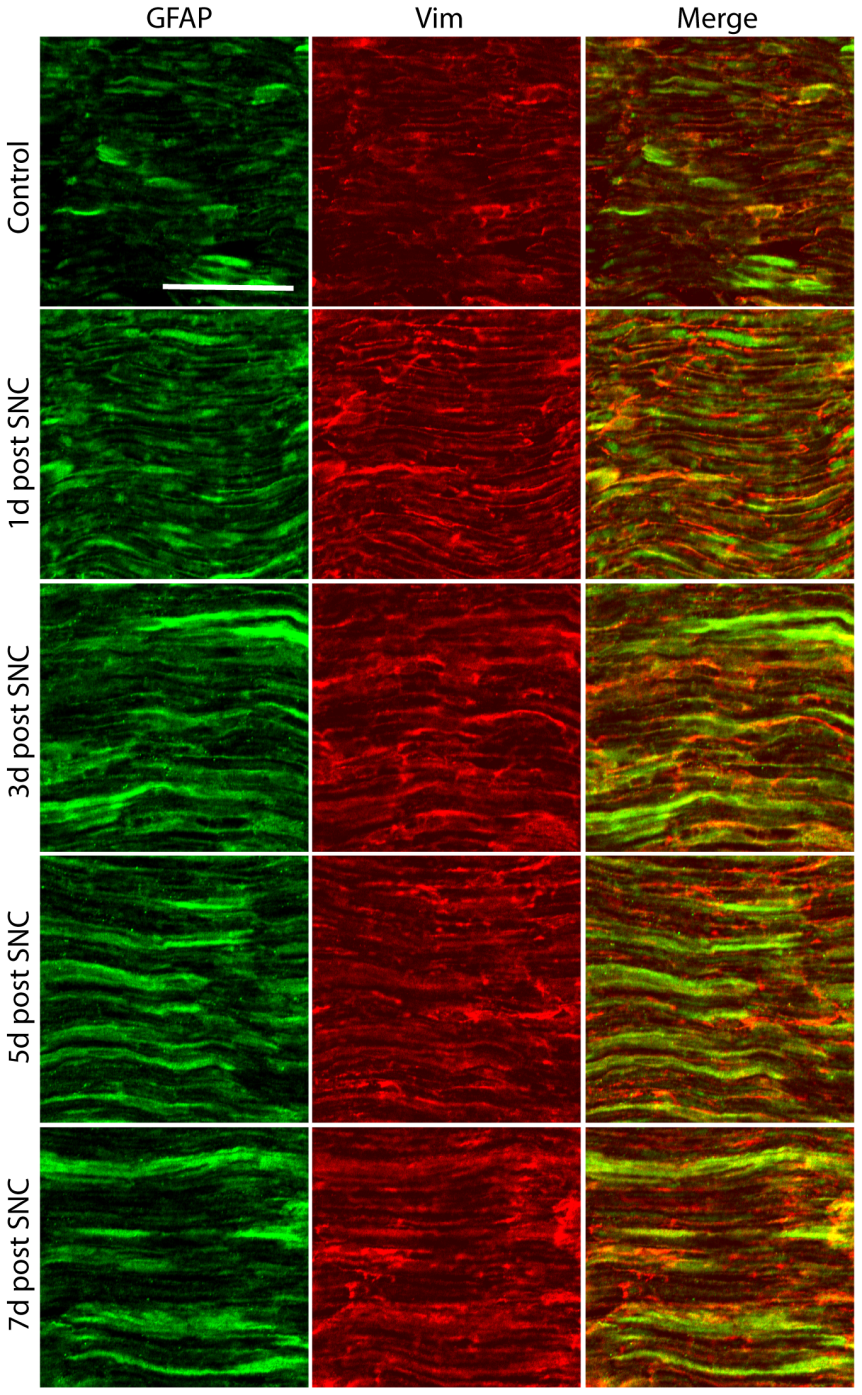
Expression of GFAP and vimentin in the lesioned sciatic nerve. Low levels of GFAP and vimentin (Vim) were detected in the uninjured nerve. GFAP and vimentin are mainly expressed by Schwann cells. An increase in both GFAP and vimentin IR is apparent from 1 day after lesion. The picture was taken as a maximal projection and settings were adjusted to optimal levels in a similar fashion for all pictures taken. Scale bar, 50 µm.

IR for GFAP and vimentin co-localized in the peripheral nerve to some extent and was upregulated in the nerve after SNC, as previously shown [23]. This upregulation was seen just proximal to the lesion site by 1 day after SNC, peaked at 3–5 days, and decreased slightly at 7 days (Fig. 2).

### Synaptic stripping in the spinal cord

**Figure 3:**
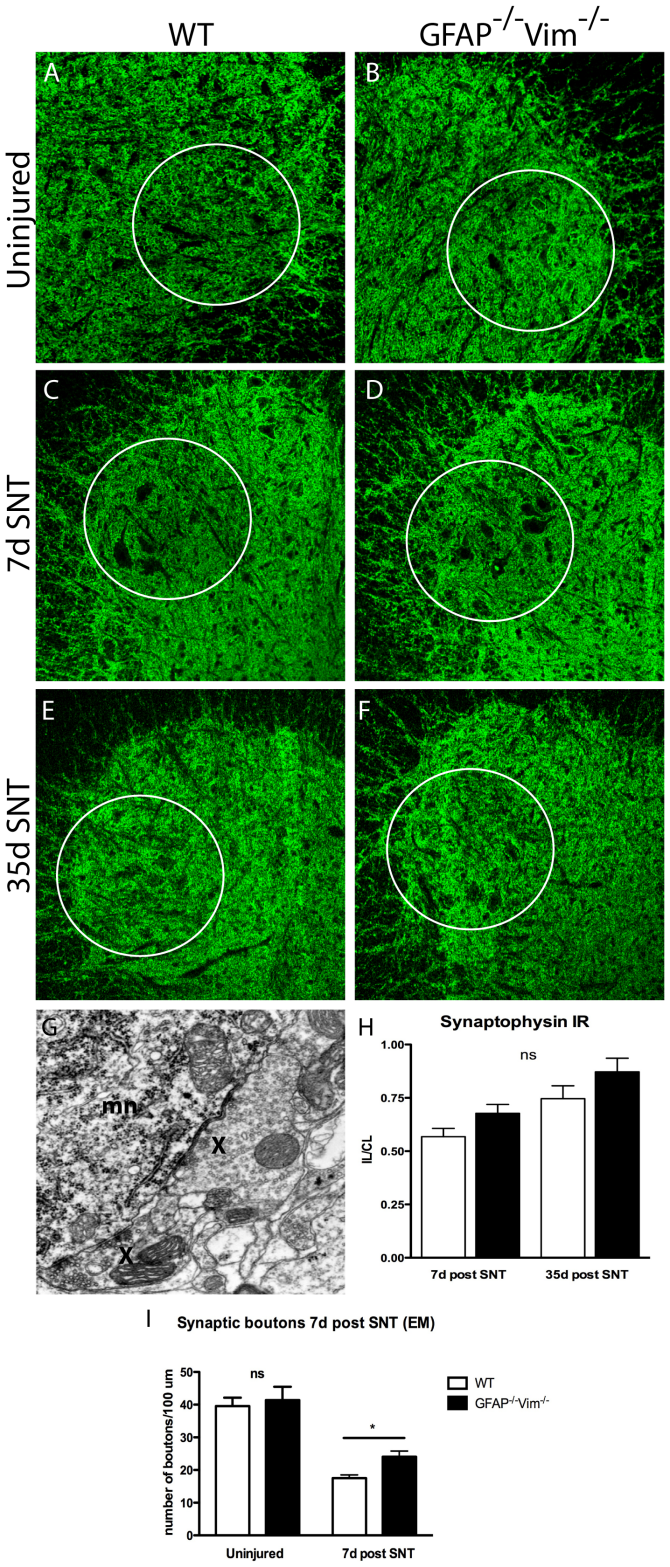
Synaptic stripping in the spinal cord 7 and 35 days after SNT. Synaptophysin IR in the ventral horn harbouring the sciatic motor pool (A-F) displayed a similar pattern in uninjured WT (n = 6) and *GFAP^–/–^Vim^–/–^* (n = 6) mice (A, B). At 7 days after SNT there was a clear decrease in synaptophysin IR in both WT (n = 6) and *GFAP^–/–^Vim^–/–^* (n = 6) mice on the ipsilateral side (C, D) compared to the contralateral side (not shown). At 35 days after SNT, synaptophysin IR had increased compared to 7 days after SNT (E, F), but no significant difference between the strains were seen at any of the two time points (H). A representative electron microscopic picture shows a motoneuron (mn) and two apposing synaptic terminals (x), (G). Seven days after SNT, electron microscopy revealed fewer boutons/100 µm of soma membrane length on the injured motoneurons in WT (n = 3) compared to *GFAP^–/–^Vim^–/–^* (n = 3) mice (I). Error bars indicate SEM. * p<0.05, ns =  non-significant. Scale bar, 200 µm.

Since axon transection has been the preferred lesion model for studying synaptic stripping of motoneurons [6]-[9], [24], we used SNT for semiquantative assessment of synaptophysin IR in lesioned motor nuclei and a quantitative ultrastructural analysis of the synaptic inputs to the cell soma of lesioned motoneurons. We first investigated the extent of synaptic stripping by assessing synaptophysin IR, in uninjured spinal cords, 1 week after SNT, when synapses are removed, and at 35 days, when synapses are being re-established. At neither time point did we observe any difference between *GFAP^–/–^Vim^–/–^* and WT mice, although there was a trend toward higher synaptophysin levels in *GFAP^–/–^Vim^–/–^* mice (Fig. 3 A-D). Seven days after SNT, *GFAP^–/–^Vim^–/–^* and WT mice had lost synapses on the somata of injured motoneurons, as shown by ultrastructural analysis; however, *GFAP^–/–^Vim^–/–^* mice had approximately 35% more boutons covering the injured somata (p<0.05 vs. WT). No difference was seen between uninjured *GFAP^–/–^Vim^–/–^* and WT mice (Fig. 3E).

### Functional recovery is slightly delayed but complete in *GFAP^–/–^Vim^–/–^* mice

Next we assessed functional recovery following regeneration after peripheral nerve injury. For this purpose, we used the SNC lesion, which provides a more controlled and reproducible model for axon regeneration than SNT and allows more or less complete functional recovery [25], [26]. Grip strength, foot faults, toe spread, and intermediary toe spread were assessed 6 days after injury and then every 7 days.

**Figure 4:**
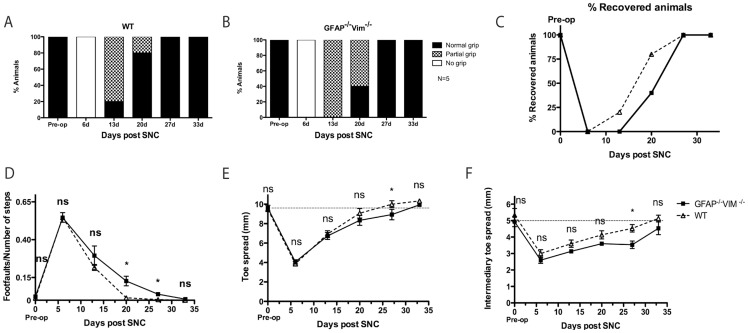
Long-term recovery of function after SNC. (A–C) Grip strength. Grip strength was complete in 20% of WT (n = 5) and none of the *GFAP^–/–^Vim^–/–^* (n = 5) at 13 days, in 80% of WT and 40% of *GFAP^–/–^Vim^–/–^* at 20 days, and in all mice at 27 days. (D) Footfaults, WT mice performed better than *GFAP^–/–^Vim^–/–^* mice at 20 and 27 days after SNC, but no difference was seen at 33 days. (E, F) With respect to toe spread (E) and intermediary toe-spread (F), WT mice had a better recovery at 27 days after SNC; however, at 33 days, all mice had recovered to preoperative levels. Error bars indicate SEM. *p <0.05 (unpaired *t* test), ns =  non-significant.

Six days after SNC, WT and *GFAP^–/–^Vim^–/–^* mice had no grip function. At 13 days, grip strength was partial in 4 of 5 WT mice and complete in 1, but was partial in all 5 *GFAP^–/–^Vim^–/–^* mice. At 20 days, grip strength was complete in 4 of 5 WT mice and in 2 of 5 *GFAP^–/–^Vim^–/–^* mice. After 27 days, both strains had full grip strength (Fig. 4 A-C).

Foot fault frequency (Fig. 4D) was about 60% in *GFAP^–/–^Vim^–/–^* and WT mice at 7 days and was greater in *GFAP^–/–^Vim^–/–^* mice than in WT mice at 20 and 27 days. At 34 days, all mice in both groups had recovered completely. Analysis of toe spread and intermediary toe spread showed a similar response in WT and *GFAP^–/–^Vim^–/–^* mice, with both groups reaching a full recovery (Fig. 4 E,F).

### Morphological analysis of regeneration in the sciatic nerve

**Figure 5:**
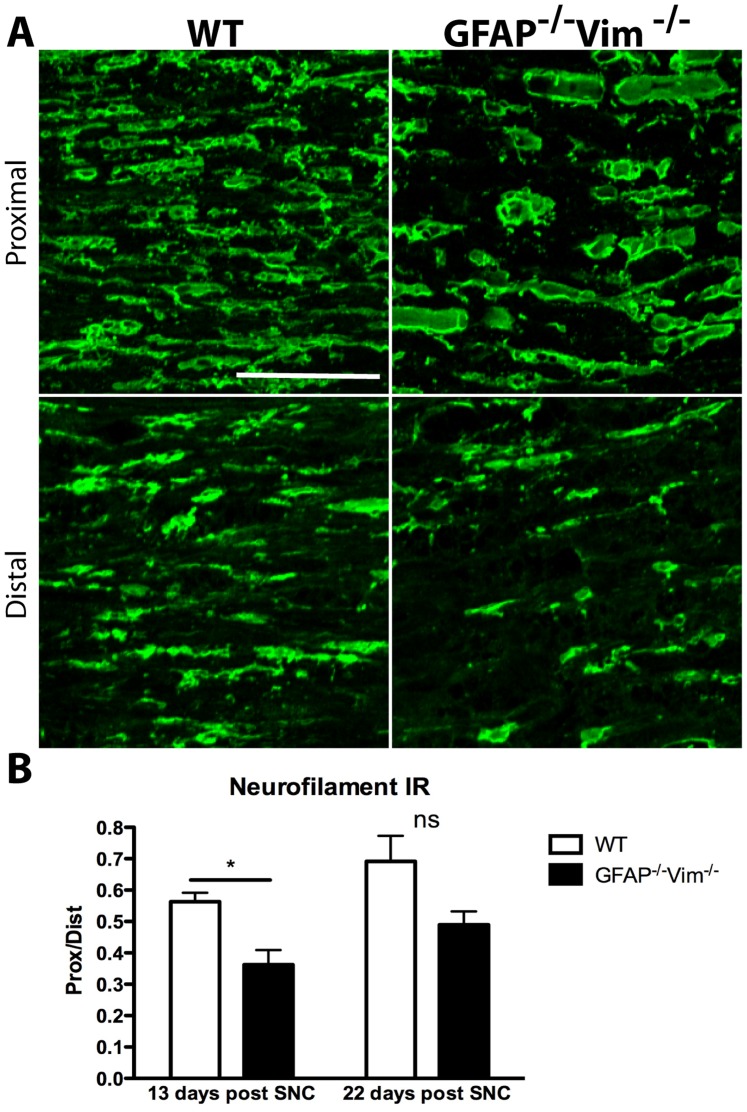
Neurofilament IR in the lesioned nerve. (A) Neurofilament IR in axons distal and proximal to the lesion site 13 days after SNC. (B) Neurofilament IR levels recovered more slowly in *GFAP^–/–^Vim^–/–^* (n = 5) than WT (n = 5) mice. Error bars indicate SEM. *p<0.05 (one-way ANOVA), ns =  non-significant. Scale bar, 50 µm.

Next, in the peripheral nerve we assessed the histological correlates to the delay in functional recovery in *GFAP^–/–^Vim^–/–^* mice. First, we assessed peripheral regrowth of axons by quantifying the IR for neurofilaments (intermediate filament proteins of neurons) in axons distal and proximal to the lesion site. At 13 days after SNC, the distal/proximal IR ratio was 0.56±0.03 (mean ± SEM) in WT mice and 0.36±0.05 in *GFAP^–/–^Vim^–/–^* mice (Fig. 5). At 22 days, the ratio was 0.69±0.08 in WT mice and 0.49±0.04 in *GFAP^–/–^Vim^–/–^* mice (P  =  NS) (Fig. 5).

### Reappearance of Schwann cell S100 IR and remyelination

**Figure 6:**
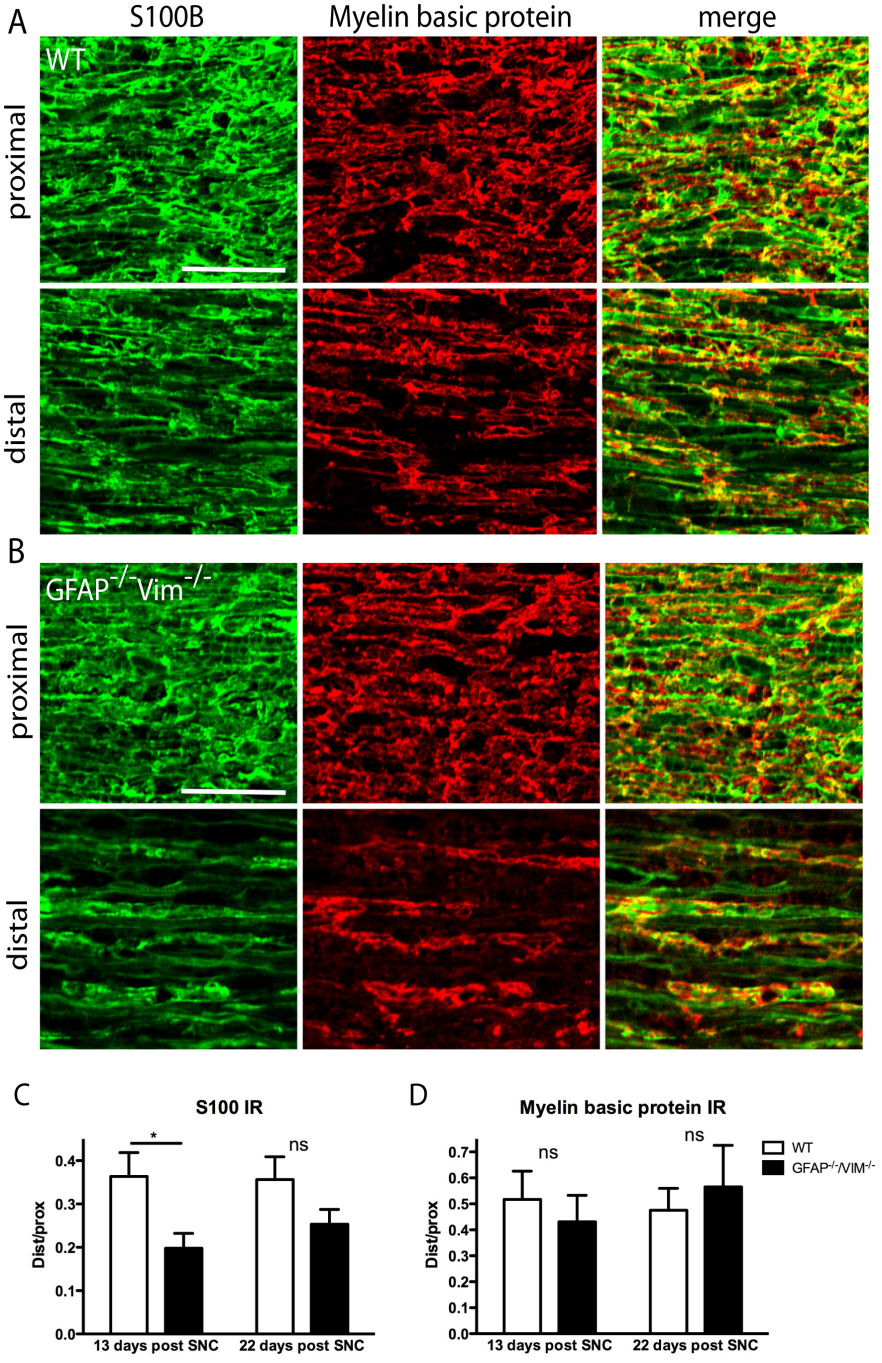
Schwann cell IR and remyelination in the injured sciatic nerve. At 13 days after SNC, we measured S100 (left column) and myelin basic protein (MBP, middle column) IR immediately proximal and distal to the lesion site. The distal/proximal ratio for both S100 and MBP IR was reduced. For S100, this decrease was more prominent in *GFAP^–/–^Vim^–/–^* mice (B, column one) than in WT mice (A), quantified in (C). We observed a decrease in MBP IR ratio both for WT and *GFAP^–/–^Vim^–/–^* mice but did not detect any difference between the two groups of mice (D). Error bars indicate SEM. *p<0.05 (one-way ANOVA), ns =  non-significant. Scale bar, 50 µm.

Normally, GFAP is present in nonmyelinating Schwann cells, and vimentin is present in both nonmyelinating and myelinating Schwann cells [17], [18]. Since Schwann cells in the *GFAP^–/–^Vim^–/–^* mice could have been primarily affected by the absence of their intermediate filament system, we assessed expression of S100 (an established marker for normal mature Schwann cells) distal and proximal to the lesion. At 13 days after SNC, the distal/proximal IR ratio was 0.20 ± 0.03 in *GFAP^–/–^Vim^–/–^* mice and 0.36 ± 0.06 in WT mice (p<0.05). At 22 days, only a nonsignificant trend remained (Fig. 6C). These findings suggest an altered response of Schwann cells in *GFAP^–/–^Vim^–/–^* mice. Next, to examine the extent of remyelination, we used antibodies to the myelin marker myelin basic protein (MBP). There were no differences between the two groups of mice (Fig. 6D).

### Reinnervation of motor endplates in the gastrocnemius muscle

**Figure 7:**
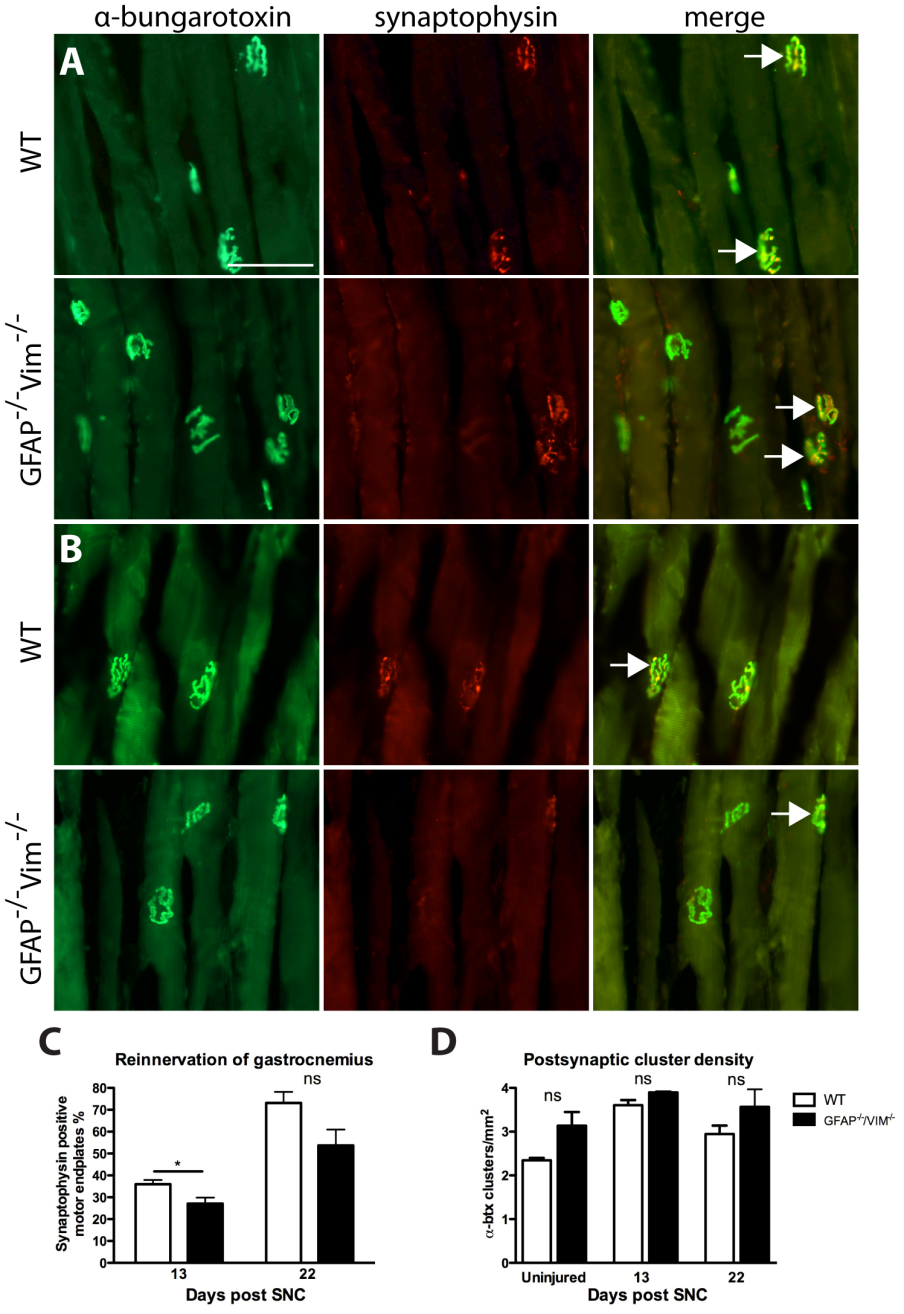
Reinnervation of the gastrocnemius muscle. At 13 days after SNC, the number of reinnervated postsynaptic clusters (i.e. synaptophysin (red, middle column) and α-bungarotoxin (green, left column) -positive clusters, and merged in right column) was higher in WT mice (n = 5) than in *GFAP^–/–^Vim^–/–^* mice (n = 5) (A and C). Arrows indicate reinnervated postsynaptic sites. At 22 days, there was no statistical difference between the two groups (B, C). The number of postsynaptic sites was similar in WT and *GFAP^–/–^Vim^–/–^* mice. In D, the postsynaptic sites were quantified on α-bungarotoxin stained sections from uninjured mice, and from lesioned mice 13 and 22 days post SNT. No difference was seen at any of the time points. Error bars indicates SEM. *p<0.05 (one-way ANOVA), ns =  non-significant. Scale bar, 100 µm.

Next, we investigated the degree of reinnervation of the denervated muscle. At 13 days after SNC, double staining for the postsynaptic marker α-bungarotoxin and the presynaptic marker synaptophysin revealed a difference in the fraction of reinnervated motor endplates: 36.0 ±1.9% in WT mice vs 27.9 ±2.5% in *GFAP^–/–^Vim^–/–^* mice (p<0.05) (Fig. 7A, C). At 22 days, no difference was seen, although the tendency persisted (Fig. 7B-C). The density of postsynaptic clusters did not differ between WT and *GFAP^–/–^Vim^–/–^* mice at 13 or 22 days (Fig. 7D).

## Discussion

Here we investigated the effect of sciatic nerve lesion on nerve regeneration and synaptic stripping in mice deficient in GFAP and vimentin, the intermediate filament proteins that are upregulated in astrocytes and Schwann cells after injury. Ultrastructural analysis of the synaptic input to the cell bodies of axotomized motoneurons 7 days after axotomy showed a less prominent removal of synaptic boutons in *GFAP^–/–^Vim^–/–^* than in WT mice. Thirteen days after sciatic nerve crush, the fraction of reinnervated motor endplates on muscle fibers of the gastrocnemius muscle was reduced in *GFAP^–/–^Vim^–/–^* mice. Axonal regeneration and functional recovery in *GFAP^–/–^Vim^–/–^* mice was complete, albeit somewhat delayed. These findings imply that the absence of GFAP and vimentin in glial cells does not affect the ultimate outcome of sciatic nerve lesion, but does affect the dynamics of the response.

Is there a causal link between the reduced removal of synapses from axotomized motoneurons and slower functional recovery after nerve lesion in *GFAP^–/–^Vim^–/–^* mice? It is unclear whether elimination of synapses from injured motoneurons after peripheral nerve injury is important for the survival and axonal regeneration of these cells. On the one hand, uncoupling the motoneuron from neuronal activity could promote recovery by allowing the metabolic machinery to concentrate on the rebuilding processes [27]–[29]. Synapse elimination may also facilitate adaptive synaptic plasticity. On the other hand, functional deficits after peripheral nerve lesion seem to be directly related to synapse elimination, as the stretch reflex from reinnervated muscles is completely lost [30], due to a loss of primary afferent terminals on motoneurons after peripheral nerve injury [31]. The answer to this controversy may depend on the functional properties of the synapses removed. Indeed, in mice deficient in MHC class Ia, increased removal of inhibitory synapses is associated with impaired axonal regeneration [9]. Moreover, in mice deficient in the complement protein C3, complement-mediated synapse elimination after sciatic nerve lesion preferentially targets inhibitory synapses, and selective sparing of inhibitory synapse in C3-deficient mice is associated with faster axonal regeneration and functional recovery [32]. By implication, selective sparing of excitatory synapses could lead to slower functional recovery. Whether this is the case in *GFAP^–/–^Vim^–/–^* mice remains to be determined.

The delayed recovery in *GFAP^–/–^Vim^–/–^* mice could also be explained by other mechanisms in the peripheral nerve or at neuromuscular junction. In support of this possibility, Schwann cell proliferation is impaired and nerve regeneration is delayed in *GFAP^–/–^* mice [16]. During the regenerative phase in our *GFAP^–/–^Vim^–/–^* mice, the distal stump of the lesioned sciatic nerve showed decreased IR both for S100, a marker of mature myelinating and nonmyelinating Schwann cells [33], [34] and for axonal markers and contained fewer synaptophysin-positive motor end plates in the reinnervated muscle. Thus, the response of Schwann cells is altered in the absence of GFAP and vimentin in glial cells and is associated with impaired axonal regeneration and muscle reinnervation. Schwann cells are important not only in the regeneration of peripheral nerve axons, but also in the formation of new neuromuscular junctions, reinnervation of postsynaptic sites [35], and even in the maintenance of stable neuromuscular junctions [19]. Since remyelination was not affected in the *GFAP^–/–^Vim^–/–^* mice, the delayed functional recovery could be caused by reduced ability of Schwann cells to proliferate and guide the outgrowing axons [2], [36], rather than by impaired remyelination.

After neurotrauma, *GFAP^–/–^Vim^–/–^* mice show attenuated reactive gliosis, reduced glial scar formation, slower healing, and increased loss of neuronal synapses [10], [11], [37]. They also show a faster progression of neurodegenerative diseases, such as Alzheimer’s disease [13] or Batten disease [14]. After ischemic stroke, *GFAP^–/–^Vim^–/–^* mice develop larger infarctions than WT mice [12]. The intermediate filament system of astrocytes has been linked to astrocyte motility [38], intracellular trafficking of vesicles [39]–[41], viscoelastic properties of astrocytes [42], elimination of reactive oxygen species [43] and volume regulation in response to hypo-osmotic stress [44]. However, attenuated reactive gliosis seems to bring some clear benefits that provide a rationale for future therapeutic modulation of reactive gliosis to improve the regenerative response. Attenuation of reactive gliosis after neurotrauma in *GFAP^–/–^Vim^–/–^* mice allows better regeneration of axons [45] and synapses [11] and improves functional recovery after spinal cord injury [15]. In *GFAP^–/–^Vim^–/–^* mice, retinal grafts are better integrated [46], differentiation of transplanted neural stem cells into neurons and astrocytes is enhanced [47], and hippocampal neurogenesis is increased under physiological conditions [48], [49] and after neurotrauma [49]. Thus, the benefits of reactive gliosis in the acute phase of CNS injury might be balanced against the restricted regenerative potential later on. Here we show that in the peripheral nervous system, the absence of the intermediate filament system in astrocytes and Schwann cells slightly slows the response to the injury without affecting the ultimate outcome. These findings imply that the consequences of astrocyte reactivity and reactive gliosis in the peripheral nervous system and CNS are different. While attenuation of reactive gliosis achieved by genetic ablation of GFAP and vimentin allows better regeneration in the CNS, possibly by supporting a prolonged healing process [50], [51], in the peripheral nervous system, where complete regenerative response can be achieved, it only slows down its dynamics. Thus, the biological role of GFAP and vimentin seems to relate to the speed and the intensity of the response to injury by astroglial cells. While this response is highly useful at the acute stage of injury, it imposes limits on the extent of regeneration in the CNS and also affects the regeneration dynamics in the peripheral nervous system. The molecular mechanisms that are involved remain to be elucidated.

In summary, this study provides evidence that the absence of GFAP and vimentin leads to reduced synaptic elimination from spinal cord motoneurons after axon lesion, reduced ability of Schwann cells to guide and support regenerating axons, slower formation of new neuromuscular synaptic contacts, and slightly delayed but complete recovery. Thus, activation of astrocytes and Schwann cells plays a role in axonal regeneration after peripheral nerve injury.
